# Safety of upadacitinib in Latin American patients with rheumatoid arthritis: an integrated safety analysis of the SELECT phase 3 clinical program

**DOI:** 10.1007/s10067-023-06513-y

**Published:** 2023-01-30

**Authors:** Adriana Maria Kakehasi, Sebastião Cezar Radominski, Marcos Daniel Baravalle, Fedra Consuelo Irazoque Palazuelos, Conrado Garcia-Garcia, Maysa Silva Arruda, Marco Curi, John Liu, Meihua Qiao, Patricia Velez-Sanchez, Juan Ignacio Vargas

**Affiliations:** 1 Hospital das Clínicas, Universidade Federal de Minas Gerais, Belo Horizonte, MG Brazil; 2 Universidade Federal Do Paraná, Curitiba, PR Brazil; 3 Instituto Médico Strusberg, Córdoba, Argentina; 4 CINTRE (Centro de Investigación y Tratamiento Reumatológico SC), Mexico City, Mexico; 5 Rheumatology Unit, Hospital General de México “Dr. Eduardo Liceaga”, Mexico City, Mexico; 6 AbbVie Farmacêutica Ltda, São Paulo, Brazil; 7 AbbVie, North Chicago, IL USA; 8 Centro de Investigación en Reumatología Y Especialidades Medicas SAS (CIREEM SAS), Bogota, Cundinamarca Colombia; 9 Quantum Research, Puerto Varas, Chile

**Keywords:** Janus kinase inhibitor, Latin America, Rheumatoid arthritis, Safety, Upadacitinib

## Abstract

**Introduction/objectives:**

Rheumatoid arthritis (RA) is a chronic autoimmune disease characterized by ongoing inflammation and degradation of synovial joints. The oral JAK inhibitor, upadacitinib, is approved for RA. We conducted an integrated safety analysis of upadacitinib 15 mg once daily (QD) in patients from Latin America (LATAM) versus the rest of the world (RoW).

**Methods:**

Treatment-emergent adverse events (AEs) and laboratory data from six phase 3, randomized controlled trials, adjusted for upadacitinib 15 mg QD use in RA, were analyzed.

**Results:**

Overall, 3209 patients received upadacitinib 15 mg QD for 7024 patient-years (PY). LATAM patients (*n* = 725) had a mean upadacitinib exposure of 1518 PY. Baseline characteristics were generally similar between LATAM and RoW populations. AE rates (including serious/opportunistic infections, tuberculosis, and herpes zoster) and deaths were comparable between populations. LATAM patients had lower serious AE rates per 100 PY (9.4 vs 14.0 E/100 PY) and discontinuation-related AEs (3.9 vs 6.0 E/100 PY) versus RoW. Rates of cardiovascular events were low (≤ 0.5 E/100 PY) and similar between populations. Malignancies, excluding non-melanoma skin cancer, were less common in the LATAM population versus RoW (0.2 vs 1.0 E/100 PY). Laboratory abnormalities were similar between populations, with decreases in hemoglobin, lymphocyte, and neutrophil counts, and elevations in liver enzymes and creatine phosphokinase. Mean change from baseline in low- and high-density lipoprotein cholesterol was generally comparable between LATAM and RoW populations.

**Conclusion:**

Upadacitinib 15 mg QD demonstrated a consistent safety profile across LATAM and RoW patient populations, with no new safety risks observed.

**Trial registration numbers:**

SELECT-EARLY, NCT02706873; SELECT-NEXT, NCT02675426; SELECT-COMPARE, NCT02629159; SELECT-MONOTHERAPY, NCT02706951; SELECT-BEYOND, NCT02706847; SELECT-CHOICE, NCT03086343.

**Supplementary information:**

The online version contains supplementary material available at 10.1007/s10067-023-06513-y.

**Table:** 

**Key Points** *• This study assessed the safety of upadacitinib 15 mg in patients with rheumatoid arthritis from Latin America compared with patients from the rest of the world participating in the SELECT phase 3 program.* *• Safety was generally similar between the two groups, including serious and opportunistic infections, tuberculosis, herpes zoster, and cardiovascular events. Malignancy, major adverse cardiovascular events, and venous thromboembolic events were infrequent in the Latin American patients.* *• Consistent with the known safety profile of upadacitinib, serious infections, herpes zoster, and laboratory abnormalities were the most common safety events in the Latin American population.*	

## Introduction

Rheumatoid arthritis (RA) is a chronic autoimmune disease characterized by ongoing inflammation and degradation of the synovial joints, with an estimated global prevalence of 0.46% [1]. However, there may be a higher prevalence of RA in the Latin American (LATAM) population, which has been reported to range from 0.4 to 1.6% [2]. Compared with the rest of the world (RoW), LATAM patients with RA show a higher ratio of females to males (5.2:1 vs 3:1), an earlier age of onset, and a poorer prognosis due to genetic and epidemiologic differences [2–5]. In the RoW, RA accounts for 0.13% of world disability-adjusted life years; in a study investigating RA in five LATAM countries (Brazil, Colombia, Venezuela, Argentina, and Mexico), it was found that these figures are higher in the LATAM population (0.16%, 0.21%, 0.24%, 0.16%, and 0.30%, respectively), indicating a higher burden of disease [6]. This may result from challenges in the management of RA in LATAM countries, such as delays in referral, limited resources, and lack of education, as well as a relatively high prevalence of infectious diseases such as tuberculosis (TB) and malaria [7]. Guidelines for treating LATAM patients with RA typically recommend the use of conventional synthetic disease-modifying anti-rheumatic drugs (csDMARDs) as a first-line treatment and biologic disease-modifying anti-rheumatic drugs (bDMARDs) or Janus kinase inhibitors (JAKis) as a second-line treatment [2, 4, 8–10].

Upadacitinib is an oral JAKi with selectivity for JAK1 over JAK2, JAK3, and tyrosine kinase 2 [11]. Upadacitinib 15 mg once daily (QD) has been recently approved in several LATAM countries for adult patients with moderately to severely active RA [12–17]. The efficacy and safety of upadacitinib have been studied in patients with moderately to severely active RA in the six SELECT phase 3, randomized clinical trials [18–23]. We recently published safety data on upadacitinib in the global population of patients with RA participating in the SELECT phase 3 studies [24], which demonstrate an acceptable safety profile of upadacitinib 15 mg QD in the treatment of moderately to severely active RA, with no new safety risks observed. More recently, publication of long-term safety and efficacy results of upadacitinib vs adalimumab over 3 years, in the ongoing long-term extension (LTE) of the SELECT-COMPARE study, has shown that the safety profile of upadacitinib was consistent with previous study-specific and integrated safety reports [25]. Here, we report a descriptive, integrated analysis comparing safety in patients from LATAM countries (Chile, Argentina, Brazil, Mexico, Guatemala, and Colombia) vs patients from the RoW participating in the SELECT phase 3 studies in RA and receiving the approved dose of upadacitinib (15 mg QD).

## Methods

### Studies and patients

Safety data were pooled from the upadacitinib global phase 3 development program for RA, which comprises SELECT-EARLY, SELECT-NEXT, SELECT-COMPARE, SELECT-CHOICE, SELECT-MONOTHERAPY, and SELECT-BEYOND (Supplementary Table 1) [18–23]. These studies evaluated upadacitinib as monotherapy or in combination with csDMARDs in patients aged ≥ 18 years with moderately to severely active RA (≥ 6 swollen and ≥ 6 tender joints, and high-sensitivity C-reactive protein ≥ 3 mg/L at screening [≥ 5 mg/L in SELECT-COMPARE and SELECT-EARLY]) who met the 2010 American College of Rheumatology/European League Against Rheumatism classification criteria [26]. Upadacitinib was administered as a monotherapy (SELECT-MONOTHERAPY, SELECT-EARLY), or in combination with methotrexate (MTX; SELECT-COMPARE) or csDMARDs, including MTX (SELECT-CHOICE, SELECT-BEYOND, and SELECT-NEXT). The studies included MTX-naïve patients (SELECT-EARLY) and those with inadequate response or intolerance to csDMARDs (SELECT-NEXT, SELECT-COMPARE, SELECT-MONOTHERAPY) or bDMARDs (SELECT-BEYOND, SELECT-CHOICE). Patients were tested for TB at screening; those with latent TB could be enrolled after initiating appropriate prophylactic treatment.

Of the six SELECT phase 3 trials in RA, LATAM patients were enrolled in every study except SELECT-BEYOND. In this analysis, raw safety data for all patients from six LATAM countries (Chile, Argentina, Brazil, Mexico, Guatemala, and Colombia) who received at least one dose of upadacitinib 15 mg QD were pooled and analyzed. This population was compared with the RoW population (with LATAM patients excluded), which included patients from North America, Eastern and Western Europe, and Asia who received at least one dose of upadacitinib 15 mg QD during any of the six SELECT phase 3 trials in RA. Patients who switched to upadacitinib from placebo (PBO), adalimumab (ADA), MTX, or abatacept (ABA) were included in the upadacitinib analysis set from the start of upadacitinib treatment. Data from patients receiving PBO, ADA, MTX, and ABA were not included in this analysis due to the small sample size in the LATAM population. Each phase 3 study had its own LTE period, where patients were followed up for a period of up to 10 years. The data cut-off date was June 30, 2020.

All studies were conducted according to the International Council for Harmonization of Technical Requirements for Pharmaceuticals for Human Use guidelines, applicable regulations and guidelines governing clinical study conduct, and the Declaration of Helsinki. Study-related documents were reviewed and approved by independent ethics committees and institutional review boards. All patients provided written informed consent before participation in the study.

### Safety assessments

Adverse events (AEs) were coded using the Medical Dictionary for Regulatory Activities (MedDRA) version 19.1. The severity of AEs was assessed based on Outcome Measures in Rheumatology (OMERACT) criteria. Grades 3 and 4 laboratory abnormalities were also determined by OMERACT criteria, except for creatine phosphokinase (CPK) and serum creatinine, which were based on the National Cancer Institute’s Common Toxicity Criteria v4.03. AEs of special interest (AESIs) were selected due to their higher prevalence among RA populations, as a customary concern for immunomodulators, or because they were labeled/emerging risks with advanced RA therapies, including JAKis. A treatment-emergent adverse event (TEAE) was defined as an AE with onset on or after the first dose of upadacitinib 15 mg QD, and no more than 30 days after the last dose of upadacitinib 15 mg QD. TEAEs were used in the analysis, unless specified otherwise. Laboratory data presented were evaluated until at least week 84.

An independent Cardiovascular Adjudication Committee blindly adjudicated all deaths and potential cardiovascular (CV) events, including potential arterial and venous thromboembolic events (VTEs). Major adverse CV events (MACE) included CV death, non-fatal myocardial infarction, and non-fatal stroke. VTEs included deep vein thrombosis (DVT) and pulmonary embolism (PE). Active/latent TB events and potential gastrointestinal perforations were assessed by the sponsor.

#### Statistical analysis

Baseline characteristics and exposure were summarized descriptively. TEAEs were summarized by MedDRA system organ class and preferred term. Exposure-adjusted event rates (EAERs) per 100 patient-years (PY) were summarized as events based on the treatment received at the time of each AE; multiple events occurring in the same patient were included in the numerator; 95% confidence intervals (CIs) were calculated based on the exact method for the Poisson mean [27].

## Results

### Patients and exposure

Across the studies, 3209 patients received upadacitinib 15 mg QD for a mean duration of approximately 2.2 years and exposure of 7024 PY. Of these patients, 725 were from LATAM countries with an exposure of 1518 PY and mean exposure duration of 2.1 years, and 2484 patients were from the RoW with an exposure of 5506 PY and mean exposure duration of 2.2 years. Baseline characteristics were generally similar between patients from Latin America and the RoW (Table 1), although LATAM patients were more likely to be female (87.4% vs 78.4%), were younger (50.6 [± 11.8] vs 55.4 [± 11.9] years, mean [SD]), and had a longer disease duration (9.3 vs 8.2 years, mean).

Compared with the RoW, at baseline, more LATAM patients tested positive for TB (16.6% vs 11.5%), fewer reported statin use (7.6% vs 12.6%, Table 2), and none had a history of HZ vaccination (vs 3.7%). Rates of CV risk factors were generally lower in the LATAM population compared with the RoW (Table 2), including the proportion of patients with a prior history of CV events (4.8% vs 14.0%), history of hypertension (29.5% vs 42.7%), history of tobacco/nicotine use—current and former (33.2% vs 39.5%), and elevated low-density lipoprotein cholesterol (19.3% vs 28.8%) (Table 2). The LATAM population was also more likely to have low high-density lipoprotein cholesterol (61.7% vs 56.0%) compared with the RoW.

**Table 1:** Baseline patient demographics and disease characteristics for patients with rheumatoid arthritis treated with upadacitinib 15 mg QD in the SELECT clinical trials

Characteristic, *n* (%), unless specified	LATAM^a^ (*N* = 725)	RoW (*N* = 2484)
Female	634 (87.4)	1947 (78.4)
Mean (SD) age, years	50.6 (11.8)	55.4 (11.9)
Race		
White	684 (94.3)	2100 (84.5)
Black or African American	20 (2.8)	150 (6.0)
American Indian or Alaska native	8 (1.1)	14 (0.6)
Native Hawaiian or another Pacific islander	0	4 (0.2)
Asian	1 (0.1)	190 (7.6)
Other	12 (1.7)	26 (1.0)
Geographic region		
North America	0	815 (32.8)
South/Central America	725 (100.0)	0
Western Europe	0	283 (11.4)
Eastern Europe	0	1082 (43.6)
Asia	0	143 (5.8)
Other	0	161 (6.5)
Time since RA diagnosis, years (mean, SD)	9.3 (8.3)	8.2 (8.4)
Median (range)	7.1 (0.0 to 51.1)	5.3 (0.0 to 54.2)
Concomitant csDMARDs at baseline		
None	145 (20.0)	516 (20.8)
MTX alone	505 (69.7)	1675 (67.4)
MTX and other csDMARDs	41 (5.7)	129 (5.2)
csDMARDs other than MTX	34 (4.7)	164 (6.6)
Prior history of herpes zoster	12 (1.7)	54 (2.2)
Prior history of herpes zoster vaccination	0	91 (3.7)
Prior history of VTE	11 (1.5)	42 (1.7)
Prior history of CV events	35 (4.8)	348 (14.0)
Positive TB test at screening	120 (16.6)^b^	284 (11.5)^c^

^a^LATAM countries include Brazil, Argentina, Mexico, Colombia, Chile, and Guatemala

^b^*n* = 724

^c^*n* = 2468

*csDMARD*, conventional synthetic disease-modifying anti-rheumatic drug; *CV*, cardiovascular; *LATAM*, Latin America; *MTX*, methotrexate; *QD*, once daily; *RA*, rheumatoid arthritis; *RoW*, rest of the world; *SD*, standard deviation; *TB*, tuberculosis; *VTE*, venous thromboembolic event

**Table 2:** CV risk factors at baseline for patients with rheumatoid arthritis treated with upadacitinib 15 mg QD in the SELECT clinical trials

CV risk factors at baseline, *n* (%)	LATAM^a^ (*N* = 725)	RoW (*N* = 2484)
Medical history of hypertension	214 (29.5)	1061 (42.7)
Diabetes mellitus	55 (7.6)	200 (8.1)
History of tobacco/nicotine use (current + former)	241 (33.2)	980 (39.5)
Elevated LDL-C (≥ 3.36 mmol/L)	140 (19.3)	714 (28.8)^b^
Low HDL-C (≤ 1.55 mmol/L)	447 (61.7)	1391 (56.0)
Statin use	55 (7.6)	314 (12.6)

^a^LATAM countries include Brazil, Argentina, Mexico, Colombia, Chile, and Guatemala

^b^*n* = 2476

*CV*, cardiovascular; *HDL-C*, high-density lipoprotein cholesterol; *LATAM*, Latin American; *LDL-C*, low-density lipoprotein cholesterol; *QD*, once daily; *RoW*, rest of the world

### Adverse events

Overall rates of AEs and deaths were similar for both populations; however, LATAM patients had fewer serious AEs (SAEs; 9.4 E/100 PY [95% CI: 7.9, 11.0] vs 14.0 E/100 PY [95% CI: 13.1, 15.1]) and AEs leading to discontinuation of the study drug (3.9 E/100 PY [95% CI: 3.0, 5.0] vs 6.0 E/100 PY [95% CI: 5.4, 6.7]). Most SAEs were pulmonary or CV in nature, although there was one case of TB, one case of sepsis, one case of multi-organ failure, two cases of HZ, and two cases of cancer (liposarcoma and glioblastoma). Treatment-emergent death rates were 0.5 E/100 PY (95% CI: 0.2, 1.0) in the LATAM population and 0.3 E/100 PY (95% CI: 0.2, 0.5) in the RoW population. Among all deaths, the most common causes were CV in nature. No trend was identified that differentiated the causes of death in LATAM vs the RoW. The rates of most AESIs including serious and opportunistic infections, TB, HZ, renal dysfunction, NMSC, gastrointestinal perforation, and CV events were similar between the LATAM and RoW groups. Malignancies excluding NMSC were reported less frequently in LATAM patients compared with the RoW (Table 3).

**Table 3:** Overview of adverse events for patients with rheumatoid arthritis treated with upadacitinib 15 mg QD in the SELECT clinical trials

E/100 PY (95% CI)	LATAM^a^ (*N* = 725; 1518 PY)	RoW (*N* = 2484; 5506 PY)
Any adverse event	220.0 (212.6, 217.6)	233.7 (229.7, 237.8)
Any serious adverse event	9.4 (7.9, 11.0)	14.0 (13.1, 15.1)
Any adverse event leading to discontinuation	3.9 (3.0, 5.0)	6.0 (5.4, 6.7)
Deaths^b^	0.5 (0.2, 1.0)	0.3 (0.2, 0.5)
Adverse events of special interest		
Infection	72.2 (68.0, 76.6)	75.6 (73.3, 77.9)
Serious infection	2.7 (1.9, 3.7)	3.4 (2.9, 3.9)
Opportunistic infection^c^	< 0.1 (0.0, 0.4)	0.3 (0.2, 0.5)
Herpes zoster	3.0 (2.2, 4.0)	3.4 (3.0, 4.0)
Active TB	< 0.1 (0.0, 0.4)	< 0.1 (0.0, 0.2)
Malignancy excl. NMSC	0.2 (0.0, 0.6)	1.0 (0.8, 1.3)
NMSC	0.2 (0.0, 0.6)	0.3 (0.2, 0.5)
Lymphoma	0	< 0.1 (0.0, 0.2)
MACE^d^ (adjudicated)	0.3 (0.1, 0.7)	0.5 (0.3, 0.7)
VTE^e^ (adjudicated)	0.4 (0.1, 0.9)	0.5 (0.3, 0.7)
Hepatic disorder	16.9 (14.9, 19.1)	10.2 (9.4, 11.1)
GI perforation (adjudicated)	0	< 0.1 (0.0, 0.2)
Anemia	4.0 (3.1, 5.2)	3.3 (2.8, 3.8)
Neutropenia	3.5 (2.6, 4.6)	2.0 (1.6, 2.4)
Lymphopenia	2.6 (1.8, 3.5)	1.4 (1.1, 1.8)
CPK elevation	7.2 (6.0, 8.7)	4.3 (3.7, 4.8)
Renal dysfunction	0.3 (0.1, 0.8)	0.3 (0.2, 0.4)

^a^LATAM countries include Brazil, Argentina, Mexico, Colombia, Chile, and Guatemala

^b^Treatment-emergent deaths

^c^Excluding TB and herpes zoster

^d^Defined as cardiovascular death, non-fatal myocardial infarction, and non-fatal stroke

^e^Includes deep vein thrombosis and pulmonary embolism events

*CI*, confidence interval; *CPK*, creatine phosphokinase; *E*, events; *GI*, gastrointestinal; *LATAM*, Latin American; *MACE*, major adverse cardiovascular events; *NMSC*, non-melanoma skin cancer; *PY*, patient-years; *QD*, once daily; *RoW*, rest of the world; *TB*, tuberculosis; *VTE*, venous thromboembolic event

EAERs for serious infections were 2.7 (95% CI: 1.9, 3.7) vs 3.4 (95% CI: 2.9, 3.9) E/100 PY in the LATAM vs RoW groups, respectively. In both groups, the most common serious infection was pneumonia. The EAER for opportunistic infection (excluding HZ and TB) in the LATAM group was < 0.1 EAERs/100 PY (95% CI: 0.0, 0.4). There was one case of bronchopulmonary aspergillosis in the LATAM population that was graded as severe and required hospitalization. The EAER for opportunistic infection (excluding HZ and TB) in the RoW population was 0.3 E/100 PY (95% CI: 0.2, 0.5). EAERs of HZ were 3.0 (95% CI: 2.2, 4.0) vs 3.4 (95% CI: 3.0, 4.0) E/100 PY in the LATAM vs RoW groups. The characteristics of HZ were consistent between the LATAM and the RoW populations, where most cases were non-serious and involved a single dermatome in both groups. In LATAM patients, there was one case of disseminated HZ and one case of HZ oticus, as reported by the investigator. Also, in the LATAM population, three events of HZ were reported as serious and required hospitalization. There was one event of serious active TB in the LATAM group vs five events in the RoW group (both < 0.1 EAEs/100 PY). There were a total of six patients who experienced seven events of coronavirus disease infection 2019 in the LATAM population, of which two were reported as SAEs and required hospitalization.

Rates of adjudicated MACE and VTEs were low and generally similar between the LATAM and RoW groups (Table 3). Regarding MACE, there were three CV deaths in the LATAM population (0.2 E/100 PY [95% CI: 0.0, 0.6]: one sudden cardiac death, one PE leading to death, and one fatal stroke) and one non-fatal stroke (< 0.1 E/100 PY [95% CI: 0.0, 0.4]). There were no events of non-fatal myocardial infarction in the LATAM group. In the RoW population, EAERs were 0.1 E/100 PY (95% CI: 0.1, 0.3) for CV death, 0.1 E/100 PY (95% CI: 0.1, 0.3) for non-fatal stroke, and 0.2 E/100 PY (95% CI: 0.1, 0.3) for non-fatal myocardial infarction. There were six VTEs in the LATAM population (three DVTs and three PEs, including one case of concurrent DVT and PE), occurring at 0.4 E/100 PY (95% CI: 0.1, 0.9) vs 0.5 E/100 PY (95% CI: 0.3, 0.7) in the RoW.

Malignancy, excluding NMSC, was lower in the LATAM group (0.2 E/100 PY; 95% CI: 0.0, 0.6) vs the RoW group (1.0 E/100 PY; 95% CI: 0.8, 1.3). Non-NMSC malignancies reported in the LATAM group were one event each of glioblastoma, metastatic squamous cell carcinoma, and myxoid liposarcoma. No cases of lymphoma were reported in the LATAM group.

Events of anemia, neutropenia, and lymphopenia were generally mild or moderate, non-serious, and few led to treatment discontinuation. Most hepatic disorders were mild or moderate; there were no cases consistent with probable drug-induced liver injury attributed to upadacitinib. Similar to the RoW data, most cases of CPK elevation in LATAM patients were asymptomatic.

A total of 35 patients experienced 65 SAEs of special interest in the LATAM population. Sixty-one of these SAEs of special interest were associated with hospitalization, but no trend between these events and hospitalization was observed. Eleven patients experienced 19 life-threatening SAEs of special interest, most of which were related to serious infections, such as pneumonia. No trend was observed between SAEs and persistent or significant disability.

### Laboratory abnormalities

In general, rates of laboratory abnormalities were similar between the LATAM and RoW populations (Tables 3 and 4). However, EAERs of neutropenia, lymphopenia, hepatic disorder, and CPK elevation were numerically higher in LATAM patients compared with the RoW (Table 3). Rates of grade 3 increases in alanine aminotransferase (6.1% vs 4.4%) and aspartate aminotransferase (3.9% vs 2.9%) were higher in LATAM patients compared with the RoW (Table 4). None of the cases were reported as an SAE and no CPK elevations led to upadacitinib discontinuation in LATAM patients. Mean change from baseline in low-density and high-density lipoprotein cholesterol was generally comparable between the LATAM and RoW populations (Supplementary Fig. 1). Grade 3 or 4 abnormalities in hemoglobin, platelets, leukocytes, neutrophils, and lymphocytes were generally similar between the groups (Table 4).

**Table 4:** Proportion of patients with potentially clinically significant hematologic and clinical chemistry values

*n*/*N* Obs (%)	LATAM^a^ (*N* = 725)	RoW (*N* = 2484)
Hemoglobin (g/L)		
Grade 3 (70 to < 80 or decreased 21 to < 30)	52/725 (7.2)	202/2476 (8.2)
Grade 4 (< 70 or decreased to ≥ 30)	20/725 (2.8)	81/2476 (3.3)
Platelets (10^9^/L)		
Grade 3 (20 to < 50)	1/725 (0.1)	1/2472 (< 0.1)
Grade 4 (< 20)	1/725 (0.1)	2/2472 (< 0.1)
Leukocytes (× 10^9^/L)		
Grade 3 (1.0 to < 2.0)	0/725	9/2476 (0.4)
Grade 4 (< 1.0)	1/725 (0.1)	4/2476 (0.2)
Neutrophils (10^9^/L)		
Grade 3 (0.5 to < 1.0)	11/725 (1.5)	29/2476 (1.2)
Grade 4 (< 0.5)	4/725 (0.6)	6/2476 (0.2)
Lymphocytes (10^9^/L)		
Grade 3 (0.5 to < 1.0)	169/725 (23.3)	633/2476 (25.6)
Grade 4 (< 0.5)	16/725 (2.2)	59/2476 (2.4)
Alanine aminotransferase (U/L)		
Grade 3 (3.0 to < 8.0 × ULN)	44/724 (6.1)	108/2475 (4.4)
Grade 4 (> 8.0 × ULN)	7/724 (1.0)	19/2475 (0.8)
Aspartate aminotransferase (U/L)		
Grade 3 (3.0 to < 8.0 × ULN)	28/724 (3.9)	73/2475 (2.9)
Grade 4 (> 8.0 × ULN)	7/724 (1.0)	11/2475 (0.4)
CPK (U/L)		
Grade 3 (> 5.0 to 10.0 × ULN)	14/724 (1.9)	51/2475 (2.1)
Grade 4 (> 10.0 × ULN)	5/724 (0.7)	22/2475 (0.9)

^a^LATAM countries include Brazil, Argentina, Mexico, Colombia, Chile, and Guatemala

*N* Obs indicates the number of patients with baseline and post-baseline values for the respective parameters

*CPK,* creatine phosphokinase;* LATAM*, Latin American; *RoW*, rest of the world; *ULN*, upper limit of the normal range

## Discussion

Based on an integrated safety analysis of patients from the SELECT clinical trial program, the overall safety profile of upadacitinib 15 mg QD appears comparable between the LATAM and the RoW populations. Rates of AEs and deaths were similar for both populations, although the rates of SAEs and AEs leading to discontinuation were lower in the LATAM patients. The most common AESIs in the LATAM population were infections, including serious infections and HZ, and laboratory abnormalities, which is consistent with the known safety profile of upadacitinib [24].

During the analysis of the long-term data with the totality of the upadacitinib phase 3 program in RA, age ≥ 75 years and smoking increased the risk of serious infection for upadacitinib [28]. However, concomitant treatment with glucocorticoids was not associated with an increased risk of infection in patients receiving upadacitinib [28]. Also, there is no clear association between infectious events and decreased neutrophil or lymphocyte counts [24].

Rates of TB are known to be higher in LATAM compared with North America and Europe [29]. In keeping with this, our analysis found that the LATAM population had higher rates of a positive TB test result at screening compared with the RoW. However, there was only one case of active TB observed in the LATAM group, and the EAER for active TB was < 0.1 E/100 PY. Although our results did not find an increased risk of TB reactivation in LATAM, TB screening is recommended before starting upadacitinib [8, 9, 12–17].

HZ is a known risk in patients with RA receiving JAKis, including upadacitinib, and a previous analysis has shown that Asian patients receiving upadacitinib were at greater risk of HZ compared with patients in other regions [30]. In contrast, rates of HZ were comparable in the LATAM population compared with the RoW, with most cases being non-serious and involving a single dermatome. No patients in the LATAM region had received vaccination against HZ at baseline; information on the efficacy of HZ vaccination in LATAM patients receiving upadacitinib therefore remains an unmet need.

While overall the risk of malignancies is not increased in patients with RA, certain malignancies (such as lung cancer, renal cancer, and lymphoma) do occur at an increased rate [31–33]. A cohort study of 84,475 patients with RA observed an increased risk of lung cancer and over double the risk of liver cancer relative to their Hispanic counterparts in the general population [34]. In an overall analysis of the SELECT studies, the rates and types of malignancy with upadacitinib were consistent with those expected in patients with RA and comparable with the rate of malignancy expected in the US population [24]. In this current analysis, LATAM patients with RA had a lower rate of malignancies (excluding NMSC) compared with the RoW (0.2 vs 1.0 E/100 PY), while NMSC rates were comparable (0.2 vs 0.3 E/100 PY). No incidences of lung or liver cancer were reported in the LATAM population.

A meta-analysis of observational studies has found a 50% increased risk of CV disease in patients with RA compared with the general population; in a large, international cohort of patients, 30% of CV events were attributable to RA characteristics, and 49% were attributable to CV risk factors [35, 36]. When comparing LATAM patients with RA with the RoW, the LATAM population had a lower history of previous CV events (4.8% vs 14.0%), and the rate of CV risk factors was generally lower in LATAM than the RoW. In addition, LATAM patients had lower use of statins at baseline compared with the RoW, although this may represent a lack of access to treatment in the LATAM region, even if they are more dyslipidemic. Nonetheless, in this safety analysis, rates of adjudicated MACE were similar between the two groups (0.3 E/100 PY in LATAM vs 0.5 E/100 PY in the RoW).

Compared with the general population, patients with RA have an increased risk of developing VTEs (0.1 to 0.4 events per 100 PY vs 0.3 to 0.7 events per 100 PY) [36–38] and VTEs are an emerging AESI in patients receiving JAKis [24]. In the pooled analysis of the SELECT studies, rates of adjudicated VTEs were similar across patients receiving upadacitinib, PBO, ADA, and MTX [24]. Both the LATAM and RoW populations showed a comparable history of VTEs (1.5% vs 1.7%) and comparable rates of adjudicated VTEs (0.4 vs 0.5 E/100 PY). However, patients receiving JAKis should be monitored carefully for signs and symptoms of VTEs and treated appropriately.

Changes in laboratory parameters, including decreases in neutrophil and lymphocyte counts and increases in transaminases and CPK, are consistent with previous JAKi findings [39, 40]. Most laboratory abnormalities were mild or moderate, non-serious, and few led to treatment discontinuation. Rates of grade 3 and grade 4 changes in laboratory parameters were generally comparable between the LATAM and RoW populations. Although the mechanism of action for the elevated CPK with upadacitinib therapy is unknown, in vitro data have suggested that this CPK elevation associated with JAKis may represent the restoration of myoblast differentiation [41].

One limitation of this study is that the data were pooled from six different clinical trials, and LATAM countries did not participate in the SELECT-BEYOND study. In addition, this is a post hoc analysis of randomized clinical trials that were not designed to compare LATAM and RoW data. As such, the number of LATAM patients and their overall time of exposure to upadacitinib 15 mg QD are lower than in the RoW population. This limits interpretation of the data and the identification of any differences between populations. Safety data in LATAM patients are therefore subject to varying patient populations, including differences in previous and failed treatments, comorbidities, and different concomitant therapies. In addition, the limited PBO exposure in the SELECT trials did not allow a PBO-controlled analysis of long-term safety; we were also unable to assess the safety of upadacitinib vs active comparators such as MTX, ABA, and ADA in LATAM patients due to relatively low patient numbers. Moreover, as each study had specific follow-up protocols and eligibility criteria, generalizability of these results to clinical practice may be limited. Despite these limitations, the results obtained provide valuable information about the safety of upadacitinib treatment in LATAM patients with RA.

In conclusion, based on integrated safety data from six phase 3 trials, there is a consistent safety profile with upadacitinib 15 mg QD for the treatment of RA in the LATAM population compared with the RoW. These results support an acceptable safety profile of upadacitinib 15 mg QD for the treatment of moderately to severely active RA in adult patients in LATAM.

## Supplementary information

Below is the link to the electronic supplementary material.

Supplementary file1 (DOCX 144 KB)

## Data Availability

AbbVie is committed to responsible data sharing regarding the clinical trials we sponsor. This includes access to anonymized, individual, and trial-level data (analysis data sets), as well as other information (e.g., protocols and clinical study reports), as long as the trials are not part of an ongoing or planned regulatory submission. This includes requests for clinical trial data for unlicensed products and indications. These clinical trial data can be requested by any qualified researchers who engage in rigorous, independent scientific research, and will be provided following review and approval of a research proposal and statistical analysis plan and execution of a data sharing agreement. Data requests can be submitted at any time and the data will be accessible for 12 months, with possible extensions considered. For more information on the process, or to submit a request, visit the following link: https://www.abbvie.com/our-science/clinical-trials/clinical-trials-data-and-information-sharing/data-and-information-sharing-with-qualified-researchers.html.

## Abbreviations

ABA: Abatacept
ADA: Adalimumab
AE: Adverse event
AESI: Adverse event of special interest
bDMARDs: Biologic disease-modifying anti-rheumatic drugs
CI: Confidence interval
CPK: Creatine phosphokinase
csDMARDs: Conventional synthetic disease-modifying anti-rheumatic drugs
CV: Cardiovascular
DVT: Deep vein thrombosis
EAER: Exposure-adjusted event rate
E/100 PY: Events per 100 patient-years
HZ: Herpes zoster
JAK: Janus kinase
JAKi: Janus kinase inhibitor
LATAM: Latin American
LTE: Long-term extension
MACE: Major adverse cardiovascular event
MedDRA: Medical Dictionary for Regulatory Activities
MTX: Methotrexate
NMSC: Non-melanoma skin cancer
OMERACT: Outcome Measures in Rheumatology
PBO: Placebo
PE: Pulmonary embolism
PY: Patient-years
QD: Once daily
RA: Rheumatoid arthritis
RoW: Rest of the world
SAE: Serious adverse event
TB: Tuberculosis
TEAE: Treatment-emergent adverse event
VTE: Venous thromboembolic event
