# Supplementary material for: Safety of upadacitinib in Latin American patients with rheumatoid arthritis: an integrated safety analysis of the SELECT phase 3 clinical program
Source: Clin Rheumatol. 2023 Jan 30;42(5):1249–58. doi: 10.1007/s10067-023-06513-y (PMC9886207; doi:10.1007/s10067-023-06513-y)
Supplement: Supplementary file 1 — Supplementary file1 (DOCX 144 KB) [file 10067_2023_6513_MOESM1_ESM.docx]

# Supplement to *Safety of upadacitinib in Latin American patients with rheumatoid arthritis: an integrated safety analysis of the SELECT phase 3 clinical program* by Adriana Maria Kakehasi (amkakehasi@gmail.com; Hospital das Clínicas, Universidade Federal de Minas Gerais, Belo Horizonte, MG, Brazil) and Sebastião Cezar Radominski, Marcos Daniel Baravalle, Fedra Consuelo Irazoque Palazuelos, Conrado Garcia-Garcia, Maysa Silva Arruda, Marco Curi, John Liu, Meihua Qiao, Patricia Velez-Sanchez, Juan Ignacio Vargas

**Supplementary Table 1** Study characteristics

|  | SELECT-EARLY (NCT02706873) | SELECT-NEXT  (NCT02675426) | SELECT-MONOTHERAPY  (NCT02706951) | SELECT-COMPARE (NCT02629159) | SELECT-BEYOND (NCT02706847) | SELECT-CHOICE  (NCT03086343) |
| --- | --- | --- | --- | --- | --- | --- |
| Patients | MTX-naïve | csDMARD-IR | MTX-IR | MTX-IR | bDMARD-IR | bDMARD-IR |
| Overall number of patients randomized | 947 | 661 | 648 | 1629 | 499 | 613 |
| Background treatment | — | csDMARDs | — | MTX | csDMARDs | csDMARDs |
| Arms | UPA 7.5 mg^a^  UPA 15 mg  UPA 30 mg  MTX | UPA 15 mg  UPA 30 mg  PBO | UPA 15 mg  UPA 30 mg  MTX | UPA 15 mg  ADA 40 mg  PBO | UPA 15 mg  UPA 30 mg  PBO | UPA 15 mg  ABA |
| Primary endpoint | ACR50 at week 12, and DAS28(CRP) < 2.6 at week 24 | ACR20 at week 12, and DAS28(CRP) ≤ 3.2 at week 12 | ACR20 at week 14, and DAS28(CRP) ≤ 3.2 at week 14 | ACR20 at week 12, and DAS28(CRP) < 2.6 at week 12 | ACR20 at week 12, and DAS28(CRP) ≤ 3.2 at week 12 | Change from baseline in DAS28(CRP) at week 12 |
| Duration of randomized, double-blind study period | 48 weeks | 12 weeks | 14 weeks | 48 weeks | 12 weeks | 24 weeks |
| Total duration of study | 5 years | 5 years | 5 years | 10 years | 5 years | 5 years |

^a^Japanese patients only

*ABA*, abatacept; *ACR*, American College of Rheumatology; *ACR20/50*, patients achieving ≥ 20/50% improvement in American College of Rheumatology score; *ADA*, adalimumab; *bDMARD*, biologic disease-modifying anti-rheumatic drug; *csDMARD*, conventional synthetic disease-modifying anti-rheumatic drug; *DAS28(CRP)*, Disease Activity Score in 28 joints with C-reactive protein; *IR*, inadequate response; *MTX*, methotrexate; *PBO*, placebo; *UPA*, upadacitinib


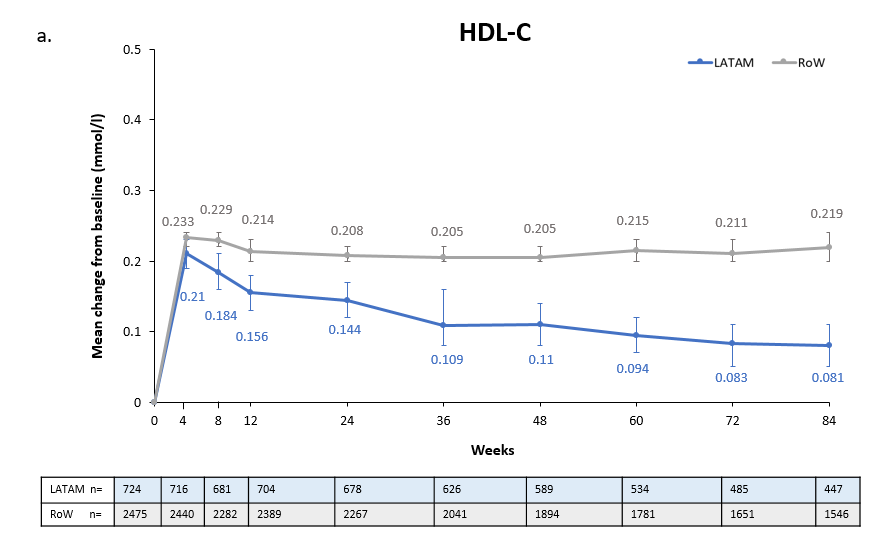
**Supplementary Figure 1** Mean change in baseline for laboratory values of HDL-C, LDL-C, and LDL/HDL ratio over 84 Weeks


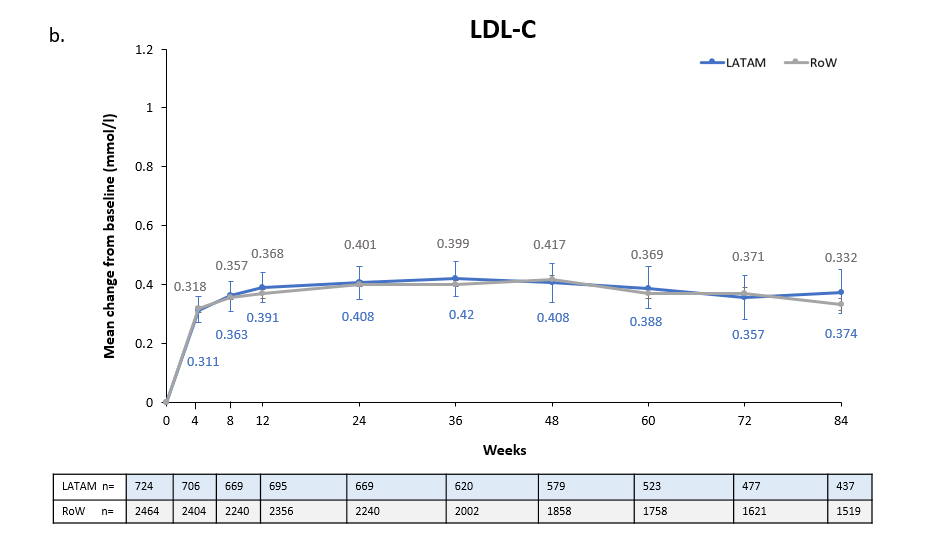


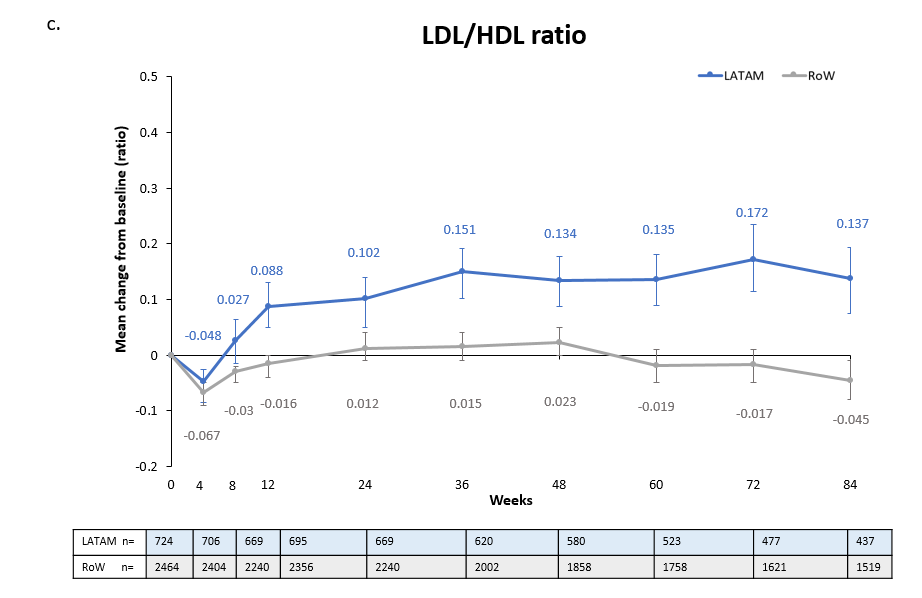


LSMeans for ANCOVA, adjusted by baseline

HDL-C, high density lipoprotein cholesterol; LDL-C, low density lipoprotein cholesterol; LS, least-squares; RA, rheumatoid arthritis; UPA, upadacitinib
